# How do national contraception laws and policies address the contraceptive needs of adolescents in Paraguay?

**DOI:** 10.1186/s12978-017-0344-z

**Published:** 2017-07-24

**Authors:** Kathya Cordova-Pozo, Sarah Borg, Andrea J. Hoopes, Alma Virginia Camacho-Hubner, Fanny Corrales-Ríos, Adriane Salinas-Bomfim, Venkatraman Chandra-Mouli

**Affiliations:** 1 South Group, Cochabamba, Bolivia; 2 Independent consultant, Oxford, UK; 3 Kaiser Permanente Washington, Bellevue, WA USA; 4 UNFPA-Latin America, Panama, Panama; 5 Ministerio de Salud Paraguay, Asuncion, Paraguay; 6 UNFPA Paraguay, Asuncion, Paraguay; 7 WHO Department of Reproductive Health and Research, Geneva, Switzerland

**Keywords:** Paraguay, Contraceptive policies and regulations, Adolescents

## Abstract

**Background:**

The main objective is to examine how the Paraguayan laws, policies and regulations (hereafter referred to as normative guidance) specifically address adolescents and their contraceptive information and service needs using a human rights analytic framework. It must be noted that this paper examines the adolescent content of national laws, policies and regulations on contraception, not how they were applied.

**Methods:**

The recommendations on “Ensuring human rights in the provision of contraceptive information and services” from the World Health Organization (WHO) were used as an analytic framework to assess current Paraguayan laws, policies and regulations. Three questions were explored: 1) whether the Paraguayan normative guidance relating to each WHO recommendation was present and specifically addressed adolescents 2) whether the normative guidance for each WHO recommendation was present but did not specifically address adolescents, or 3) whether Paraguayan normative guidance relating to each WHO recommendation was absent. This assessment led to the development of an analytic table which was used by the co-authors to generate conclusions and recommendations.

**Results:**

The analysis found specific normative guidance for adolescents relating to six out of nine WHO summary recommendations and nine out of the 24 sub-recommendations. The guidance included strategies to overcome contraceptive service barriers and to improve access for displaced populations. Further, it supported gender-sensitive counselling, quality assurance processes, competency-based training, and monitoring and evaluation of programmes.

**Conclusions:**

Paraguay’s contraception laws and policies are grounded in human rights principles. However, there are a number of aspects that need to be addressed in order to improve the quality of contraceptive provision and access for adolescents. Our recommendations include improving accessibility of contraceptive information and services, ensuring acceptability, quality, and accountability of contraceptive information and services, and promoting community and adolescent participation in contraceptive programmes and service delivery.

## Plain English summary

The purpose of this article is to examine how Paraguay’s current laws, policies and regulations specifically address adolescents and their contraceptive information and service needs using a human rights lens.

The recommendations on “Ensuring human rights in the provision of contraceptive information and services” from the World Health Organization (WHO) were used as a benchmark to assess the Paraguayan laws, policies and regulations. Three questions were explored: 1) whether the Paraguayan documents contained guidance relating to each WHO recommendation, and if so whether adolescents were explicitly named 2) whether the guidance for each WHO recommendation was present but without specifically addressing adolescents, or 3) whether the guidance for each WHO recommendation was absent.

Our analysis found specific guidance for adolescents relating to six out of nine WHO summary recommendations and nine out of the 24 sub-recommendations. The guidance included strategies to overcome contraceptive service barriers and to improve access for displaced populations. Further, it supported gender-sensitive counselling, quality assurance processes, competency-based training, and monitoring and evaluation of programmes.

Paraguay’s contraception laws, policies and regulations are grounded in human rights principles. However, there are a number of aspects that need to be addressed in order to improve the quality of contraceptive provision and access to adolescents. Our recommendations include making it easier for adolescents to obtain contraceptive information and services, making them more friendly and responsive to adolescents’ preferences, improving quality and accountability of contraceptive information and services, and promoting community and adolescent participation in contraceptive programmes and service delivery.

## Background

The rights of adolescents to health, including sexual and reproductive health (SRH) is guaranteed in the National Constitution of Paraguay and in international treaties signed and ratified by the country. The Montevideo Consensus on Population and Development, signed by Paraguay in 2013, is the most comprehensive and sound regional agreement on population and development to date. This document contains a series of inter-governmental agreements to strengthen the implementation of population and development issues beyond 2014. It contains over 120 measures targeting the eight priority areas in follow up to the Programme of Action of the United Nations International Conference on Population and Development (ICPD) held in Cairo in 1994. The Consensus was signed by 38 member countries and associate members of the United Nations Economic Commission for Latin America and the Caribbean (ECLAC) including Paraguay, thereby reiterating their joint commitment to overcoming health inequities and to ensuring gender equality and the exercise of sexual and reproductive rights. The Consensus includes important policies which ensure universal access to SRH. These policies are intended to enable individuals to exercise their sexual rights by guaranteeing the right to make informed, voluntary decisions about sexual activity and SRH. Measures to promote and safeguard SRH include access to sexuality education, effective contraception including emergency contraception, appropriate management of unwanted pregnancies, and post-abortion healthcare [1].

Under this agreement and the Millennium Development Goals, Paraguay has sought to improve policies related to SRH, especially regarding access to contraception. In order to comply with international agreements, Paraguay has approved many legislative measures in the past five years. These include a resolution that ensures the country’s obligation to provide access to quality health services that are private, confidential and non-discriminatory [2], a law that calls for the allocation of funding for reproductive health programmes and the provision of safe delivery kits [3], and a norm for humane care following abortion [4].

Paraguay has a population of approximately 6.5 million [5] of whom 20% are adolescents aged 15–19 years old. The adolescent fertility rate has fallen steadily over the past 25 years: from 92 births per 1000 women aged 15–19 in 1990, to 58 in 2014. With this decline by 37%, Paraguay has experienced one of the most substantial reductions in adolescent birth rate in the region compared to other South American countries such as Ecuador or Venezuela (12% and 18% declines, respectively) [6]. This reduction has been associated with a rising prevalence of modern contraceptive use, which increased from 48% in 1998 to 70% in 2008. Still, Paraguay has substantial levels of adolescent fertility. The adolescent fertility rate in 2004 was 78 and now is 58. According to the Demographic and Health Survey 2008, the percentage of adolescent women (15–19 years) who were pregnant or have had at least one child was almost 12% and for women aged 20–22 years old was 45.5% [7].

Despite recent reductions in the adolescent fertility rate, the burden of unintended adolescent pregnancy persists and disproportionately affects those of lower education, lower socio-economic and rural status. This has negative health effects on young mothers and their babies, as well as negative social effects, such as hindering them from continuing their education with lifelong consequences.

We sought to systematically evaluate the current policy and regulatory environment in Paraguay as it relates to adolescents’ access to contraceptive information and services. The purpose of this paper is to examine how the Paraguayan laws, policies and regulations specifically address adolescents and their contraception education and service needs within a human rights analytic framework. It must be noted that this paper examines the adolescent content of national laws, policies and regulations (hereafter referred to as normative guidance) on contraception, not how they were applied. Sound policies and strategies can contribute to improved availability, accessibility and a well-informed choice of contraception if they are effectively implemented. This will lead to a decline in unplanned/mistimed and unwanted pregnancy, and take a step further in fulfilling adolescents’ rights to contraceptive information and services.

## Methods

To examine the extent to which Paraguayan normative guidance pertaining to contraception addresses adolescents, five laws and policies were included in the analysis (Table 1). We included and analysed documents that had policy, programme or service delivery recommendations on contraception. All instances where adolescents were referenced in the listed documents were identified through careful reading of the text. Additional normative guidance documents that were reviewed for background analysis, considered for inclusion in the analysis but then excluded are also listed in Table 1.

**Table 1:** Paraguay’s laws and policies reviewed

Official title of laws and policies	Brief Title^a^
1. National Plan of Sexual and Reproductive Health 2014–2018	National Plan of SRH
2. Law 4313: To ensure funding for reproductive health programs and the provision of a kit for delivery, Ministry of Public Health and Social Welfare (2011)	SRH Funding Law
3. National Family Planning Guide and STI Management (2006)	FP/STI Guide
4. Resolution 146 on the obligation to give access to health services with quality, without discrimination, with confidentiality and guarantee of professional secrecy (2012)	SRH Quality Service Resolution
5. Adolescence Clinical Manual: integrated management of adolescents with a focus on rights (2015)	Adolescent Clinical Guideline
Other laws that were reviewed for background analysis of the legal framework on SRH:	
6. Law 2.169/03 which sets the age of majority (2003)	Age of Majority Law
7. Decree 773 for national day of family planning (2013)	National Day of Family Planning
8. \Plan of sexual reproductive health 2009–2013.	Previous National Plan of SRH
9. Law 1680/01. Code of childhood and adolescence - Convention on the rights of the child (2001)	Code of Childhood-Adolescence
10. Sanitary Code of Paraguay, Law 836 (1980)	Sanitary Code
11. Normative for humanized service in post abortion services (2012)	Humanized Post Abortion

^a^In the following sections of the paper, the normative guidance documents analysed will be referred to as ‘brief title’

After the five normative guidance documents were shortlisted, content relating to adolescents was identified and analysed by comparison with the recommendations from the World Health Organization (WHO) Guidelines, “Ensuring human rights in the provision of contraceptive information and services” [8]. These Guidelines include nine major recommendations and 24 sub-recommendations on ensuring human rights principles and practices in contraceptive delivery. The results of the analysis are classified with a colour scheme; green indicates that specific normative guidance pertaining to adolescents is present; yellow indicates that normative guidance pertaining to all populations (but not specifically adolescents) is present; gray indicates that normative guidance for that recommendation is not found in the Paraguay normative law and policies. A row with both green and yellow colours indicates that normative guidance pertaining both to all populations and to adolescents specifically is present (Table 2).

**Table 2:**
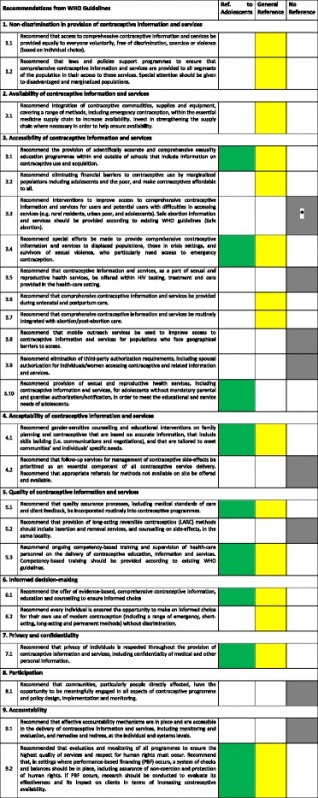
Recommendations from “Ensuring human rights in the provision of contraceptive information and services” (WHO, 2014) and comparative analysis of legislation in Paraguay

*There is normative guidance for the first part of 3.3 (i.e. for disadvantaged individuals to obtain contraceptives), including specific provisions for adolescents. Regarding the second part of 3.3, abortion is illegal in Paraguay, and so the normative guidance contains no information on safe abortion and services. However it recommends humane post-abortion care

Green = Specific normative guidance pertaining to adolescents is present

Yellow = Normative guidance pertaining to all populations (but not specifically adolescents) is present

Gray = Normative guidance for that recommendation is not present Contains no reference in the Paraguayan normative law and policies

These recommendations aim to improve the provision and utilization of contraception by examining and addressing widespread barriers to contraceptive uptake such as the non-availability of services, discriminatory practices, poor privacy and confidentiality, and lack of community participation – all based on an explicit human-rights approach. The WHO Guidelines were used to analyse the data because they provided a clear analytic framework grounded in human rights. Further, an operational version of the document is being used to guide country-level action.^1^ The recommendations have previously been used to analyse the adolescent content of South Africa’s contraception policies, strategies and guidelines by Hoopes et al. [9].

### Assessment process

The assessment process explored and analysed Paraguayan normative guidance regarding each WHO recommendation and sub-recommendation as listed in the methods section. This assessment led to the development of an analytic table (Table 2) which was used to generate conclusions and recommendations.

The analysis was a team effort and proceeded in the following manner: A political economist (K.C.P.) first reviewed the normative guidance documents to assess their content alongside the human rights analytic framework; an adolescent medicine physician and researcher (A.H.), verified the analysis of the normative guidance; a medical doctor with public health training and expertise in adolescent SRH research and programmes and a scientist at the WHO (V.C-M.), checked the outputs of the work working with a medical doctor with public health training (S.B.); and a regional technical medical adviser in SRH in the Regional Office for Latin America and the Caribbean at UNFPA (V.C.), went over the findings and strengthened them based on first-hand knowledge of the local context. The two authors from Paraguay’s Ministry of Health and UNFPA Paraguay (F.C.R. and A.S.B.) provided information and explanations on the country level context. The paper was drafted by K.C.P, and all authors contributed to the successive drafts. V.C.M and S.B. made major contributions to this. The team verified the assessment findings and generated conclusions together.

## Results

Normative guidance explicitly referring to adolescents for six out of nine summary recommendations, and nine out of 24 sub-recommendations, was identified. A detailed analysis of each recommendation and sub-recommendation is presented below, and a visual summary is included in Table 2. Beyond the normative guidance, our analysis identified elements within the *Adolescent Clinical Guideline* that addressed four out of the nine recommendations. Paraguayan national law defines adolescents as individuals 14–17 years of age [10] while the WHO defines them as as those between 10 and 19 years. There was no explicit age definition for adolescents in the normative guidance documents assessed, except for a reference to the WHO definition within the *Adolescent Clinical Guideline* [11].

### Non-discrimination in the provision of contraceptive information and services (1.1–1.2)

Regarding access to information and services (1.1), the *SRH Funding Law* establishes a constitutional right for citizens to decide when and how many children they want to bear [3]. This right can be exercised fully without discrimination on the grounds of sex, marital status, creed or location (1.2). This law and *the National Plan of SRH* establish the right of all individuals to receive SRH education with scientifically sound guidance, and adequate health services [3, 12].

Regarding laws and policies to ensure that comprehensive contraceptive information and services are provided to all segments of the population (1.2), the normative guidance calls for national programmes on SRH to be implemented as part of health service provision and education programmes with an emphasis on rights and non-discrimination. It also guarantees the provision of contraceptive methods to all [12]. The right to receive truthful, responsible and impartial information is included in the Paraguayan National Constitution (Ar. 28, PNC) (1.2). Contraceptive information and service provision are included in the *Adolescent Clinical Guideline* [11]. The *National Plan of SRH* is strongly grounded in a rights-based approach and advocates for the inclusion of all people. It includes all service delivery mechanisms and calls for links with other SRH programmes such as Human Immunodeficiency Virus (HIV) prevention, detection and care, assistance to victims of violence, and prevention of cervical and breast cancer [12] (1.2).

### Availability of contraceptive information and services (2.1)

Regarding the integration of contraceptive commodities, supplies and equipment into the essential medical supply chain (2.1), the *FP/STI Guide* calls for free contraceptives be made available to all citizens [13]. This provision includes the description of a public network for the delivery system, standards of delivery and systematic monitoring to avoid periodic shortages. The *National Plan of SRH* calls for the availability of emergency contraceptive pills, functioning condom dispensers, and counselling services to be available for 24 h every day [12]; however, there is no explicit reference to adolescents.

### Accessibility of contraceptive information and services (3.1–3.10)

The sub-recommendations on accessibility focus on barriers that reduce access to contraceptive information and services. They include poor contraceptive knowledge, lack of awareness and understanding of contraception, financial cost, distance to contraceptive distribution points, and the requirement of spousal authorization. Six of the ten sub-recommendations on accessibility address the general population, three address adolescents explicitly, and three are missing from the Paraguayan normative guidance documents.

Regarding the provision of comprehensive sexuality education (CSE) (3.1), the importance of educating clients on the risks and benefits of contraception is emphasized in the *FP/STI Guide* [13]. The *National Plan of SRH* requires adolescents be provided with CSE that addresses rights and responds to adolescents’ needs. It also requires strategies to be put in place to reach in- and- out of school adolescents via health facilities, educational institutions including public and private schools, universities and technical institutes, and the community at large [12].

In order to eliminate financial barriers (3.2), the *FP/STI Guide* calls for the provision of high quality contraceptive information and services, including free provision of contraception. Further it calls for the elimination of financial barriers to contraceptive use so that everyone can exercise their right to obtain contraceptive information and services. Based on the *SRH Funding Law*, Paraguay has a specific fund for purchasing contraceptives and other necessary supplies for contraception provision [3, 13]. The *SRH Funding Law* does not make any specific reference to adolescents but where the normative guidance states that contraception is a free service it refers to all segments of the population. (3.2).

Regarding interventions to increase access of contraceptive services to disadvantaged sections of the population (3.3), both the *National Plan on SRH* and the *FP/STI Guide* call for services that facilitate access to contraceptives for those who are disadvantaged, and for services that adequately respond to the needs of users at any hour of the day. This includes specific provisions for adolescent males and females and adult men, and strategies to increase access of SRH services to the most disadvantaged, namely those who are affected by violence, those who are disabled, HIV-positive people, or those who identify as lesbian, gay, bisexual, transgender or intersex (LGBTI) [12, 13]. It also calls for special attention to indigenous populations. Abortion is illegal in Paraguay, and so the normative guidance contains no information on safe abortion and services (3.3), with the exception that humane post-abortion care is provided in line with the approved post-abortion care guide, that includes ensuring the availability of all contraceptive methods [4].

The normative guidance in the *National Plan of SRH* explicitly calls for attention to people affected by gender-based violence (GBV), but does not do so for displaced populations, migrants or those in crisis settings (3.4) [12]. The *SRH Funding Law* requires that adolescents have access to emergency contraception and other contraceptives, particularly for those who are affected by GBV and family violence [3]. Our analysis did not find normative guidance recommending that contraceptive information and services be offered along with HIV testing and care (3.5). The *Adolescent Clinical Guideline* [11] states that all health centres could offer SRH care including contraceptives (1.1 and 2.1). All health centres can provide information, education and counselling but only specialized hospitals are responsible for providing STI and HIV preventive care.

Regarding the provision of contraceptive information and services as part of prenatal and postnatal care (3.6), the *National Plan of SRH* mandates that information and contraceptive services are available during these important moments of care. It also highlights the importance of preventing rapid repeat pregnancies in adolescents [12].

For integrating contraceptive provision within abortion care (3.7), quality, technical and personalized care are part of the required standard of care for abortion complications. Supporting the prevention of recurrence is emphasized in the *Norm for Humane Care following abortion*, but does not specifically refer to adolescents [4] (3.7).

The normative guidance documents do not address mobile services, which are required to be provided for populations who face geographic barriers to access (3.8), or the elimination of spousal or third-party authorization for individuals accessing contraceptive services (3.9).

On the elimination of parental authorization for adolescents (3.10), the *Code of Childhood-Adolescence* states that adolescents aged 14 years and older can consent to their own health services but the decision is made by their parents [14, 15]. However, the *Adolescent Clinical Guideline* [11] indicates explicitly that adolescents have the right to challenge their parents’ decisions (3.10).

### Acceptability of contraceptive information and services (4.1–4.2)

Regarding counselling and educational interventions (4.1), the *SRH Funding Law* requires that training on sexual and reproductive rights must occur in accordance with a client’s level of education, be timely, and aim to facilitate autonomy and power in decision making and reproductive responsibility [3].

Our review did not find normative guidance on follow-up services for the management of side effects of contraceptives (4.2).

### Quality of contraceptive information and services (5.1–5.3)

The *National Plan of SRH* and the *FP/STI Guide* are in line with WHO’s Medical Eligibility Criteria. The former details the characteristics, effectiveness, and correct use of each contraceptive method as a required standard of care [12, 13]. On ensuring the quality of contraceptive information services (5.1), the *National Plan of SRH* and the *FP/STI Guide* require that information be delivered according to patient choice, and that patients are treated with courtesy and consideration. However, there is no specific provision for client feedback [12, 13]. The *National Plan of SRH* requires an efficient management plan and monitoring and evaluation (M&E) strategy for the family planning programme [12].

There was no relevant normative guidance on the provision of long-acting reversible contraception methods (5.2).

The normative guidance documents address competency-based training (5.3). The *National Plan of SRH* outlines the knowledge, attitudes and skills of healthcare personnel that are needed to provide quality counselling and ensure that family planning services are rights-based and non-discriminatory. It requires that health personnel provide comprehensive care for serving adolescents, addressing the determinants of their health, and using national standards and protocols for adolescents with an emphasis on life skills, leadership, decision making, negotiation, and rights [12].

Our review did not identify guidance on supervision of health-care personnel in the delivery of these services (5.3).

### Informed decision-making (6.1–6.2)

To enable informed choice (6.1 and 6.2), the *FP/STI Guide* requires that all clients receive information on contraceptive methods (including risks and benefits) and services that can help them make fully informed decisions, including accepting or rejecting the services or requesting a referral. This informed decision-making should take into account the person’s needs, as outlined in a standardized counselling approach described in the document (6.1) [13].

There was specific normative guidance in the *National Plan of SRH* for adolescents (6.1), calling for contraceptive counselling that is culturally sensitive, and respects freedom of choice [12].

The normative guidance in the *FP/STI Guide* calls for users to be given the opportunity to adopt, change and discontinue any contraceptive method without being judged (6.2) [13]. Furthermore, the *SRH Funding Law* emphasizes the right of every woman to receive free contraceptives as per her informed decision [3].

### Privacy and confidentiality (7.1)

The *SRH Funding Law*, *SRH Quality Service Resolution*, and the *FP/STI Guide* all include normative guidance on ensuring private and high quality services and confidentiality (7.1) [2, 3, 13]. In line with the *SRH Quality Service Resolution*, health professionals must respect patient confidentiality. Furthermore, reporting of abortion complications or ongoing post abortion care must protect the confidentiality of the patient [2]. In regards to adolescents, both the *Adolescent Clinical Guideline* [11] and the *SRH Quality Services Resolution* state that young people have the right to information and access to health services, including those related to SRH, and all health professionals must respect their confidentiality. The *Code of Childhood-Adolescence* states that services and programmes for adolescents should offer professional secrecy [15] and the *Adolescent Clinical Guidelines* describes the principles of confidentiality, respect of privacy, and ethical principles [11].

### Participation (8.1)

Our review did not find normative guidance regarding participation of adolescents in policy design, implementation or monitoring (8.1).

### Accountability (9.1–9.2)

Our review did not find normative guidance recommending effective accountability mechanisms to be in place for the delivery of contraceptive information and services at the individual or systems level (9.1).

Regarding monitoring and evaluation (M&E) (9.2), the State guarantees free contraceptive services for all. The *National Plan of SRH* states that contraceptive services must be comprehensive and effective. It calls for the services to ensure respect and to observe clients’ rights. It also calls for sound M&E [12]. The guidance emphasizes monitoring of services to identify discrimination against vulnerable populations, including adolescents, indigenous people, people from less privileged socioeconomic backgrounds, people living with HIV, people living with disabilities, older adults, LGBTI people, and commercial sex workers [12].

## Discussion

The national normative guidance of Paraguay addresses many aspects of human rights in the provision of contraceptive information and services to the general population, although not consistently for adolescents (Table 2). Of the nine WHO recommendations, privacy and confidentiality (7.1) is most comprehensively addressed in the normative guidance specifically for adolescents, while the remaining WHO recommendations are only partially addressed for adolescents. The WHO recommendation for participation (8.1) is not addressed at all.

Our review identified strengths in the normative guidance for adolescents in the provision of CSE (3.1), notably a call for increasing access to those facing barriers to contraceptive services (3.3), and displaced populations (3.4). The normative guidance also refers to gender-sensitive counselling (4.1), quality assurance processes (5.1), competency-based training and supervision (5.3), informed choice (6.1), privacy and confidentiality (7.1) and M&E of programmes (9.2). The review also identified areas of weakness; Age ranges of adolescents were inconsistent across normative guidance documents, which could result in confusion among policymakers and health personnel. Table 3 contains a list of recommendations that could be considered for inclusion when laws and policies are being reformed.

**Table 3:** Potential opportunities to strengthen in Paraguay’s Contraception Legislation

Accessibility of contraceptive information and services (3):
Provide information about contraception within HIV testing, treatment and care (3.5)
Include mobile outreach services to reduce geographical barriers (3.8)
Explicitly state that adolescents do not need a third party authorization (3.9) (3.10)
Ensure acceptability (4), quality (5), and accountability (9) of contraceptive information and services:
Provide follow up services to manage contraceptive side effects (4.2)
Extend the provision of long-acting reversible contraception and removal (5.2)
Include monitoring and evaluation of programs to ensure quality and respect for human rights (9.1)
Identify best practices to ensure consistent contraceptive availability and prevent stock-outs (9.2)
Provide supervision of health-care personnel delivering services (5.3)
Promote community and adolescent participation in contraceptive programs and services: (8)
Explicitly include migrant, indigenous, poor and rural populations (8.1)
Involve adolescents and the community in different aspects of the contraceptive program (8.1)

By providing adolescents with information and notifying them that they do not need authorization from a third party, as indicated in Table 3, accessibility of contraceptive information and services can be increased. Reaching out to peri-urban and rural adolescent population can reduce geographical barriers and increase accessibility. Offering placement and removal of long-acting reversible contraceptive (LARC) devices can help to ensure acceptability and quality of contraceptive services. Adolescents need to be informed about side effects of contraceptive methods to ensure quality information and services. Also, better oversight of programmes can help to identify weaknesses and problems and ensure effective design, implementation, and M&E of programmes. In order to promote community and adolescent participation, normative guidance needs to include migrant, indigenous, poor and rural populations and guarantee their participation in different aspects of the contraceptive programme.

The analysis of the Paraguayan normative guidance using WHO’s “Guidance on Ensuring Rights in the Provision of Contraceptive Information and Services” as an analytic framework was feasible and provided valuable insights. It pointed to the strengths and weaknesses in the adolescent content of national laws, policies, strategies and guidelines on contraception.

The review builds on a similar analysis of South Africa’s national contraception policies and strategies and guidelines by Hoopes et al. [9]. Comparing the cases of Paraguay and South Africa, there are similarities and differences. Amongst the similarities, normative guidance documents from both countries are based on a human rights framework and are inclusive of adolescents in many aspects. In both countries, the normative guidance does not adequately address adolescents in relation to informed decision-making, accountability and participation. However, South Africa’s normative guidance includes provisions on non-discrimination to prevent providers from restricting contraceptive services based solely on age, while no such provision exists in Paraguay’s normative guidance. In general, this analysis of Paraguay’s normative guidance documents demonstrates that adolescents are recognized as a population with special needs, although there is room for improvement especially in addressing social and cultural factors that affect access. We believe it would be useful to extend this analysis to other countries which have signed the Montevideo Consensus, and to match the findings of the policy analysis in this paper to programme quality and coverage, and indicators of adolescent SRH, notably adolescent fertility.

The analysis in this paper was restricted to five documents related to contraceptive laws and policies, and this may represent a limitation as there are other documents pertaining to youth and SRH that may be related indirectly to contraception. These include Law 2169/03 which defines adult age [10], Law 5419 [15], with a partial reform to the civil code in regards to age for marriage, Resolution 612 on National Policy of Health 2015–2030 [16], the National Programme 2010–2015 for prevention and comprehensive care for women, girls, children, and adolescents in situations of violence [17], Decree 773 for the National Day of Family Planning [18], HIV/AIDS Prevention and Care [19], and *Law 836* or Sanitary Code [20]. Furthermore, the review was limited to a review of documents. Assessing the impact of these normative guidance documents at the service-delivery level was beyond its scope, as noted in the methods section.

To complement this analysis, it will be equally important to analyse how and to what extent the implementation of the laws, policies and guidelines has occurred. Incomplete implementation could lead to barriers in terms of accessibility, acceptability or quality perception for adolescents.

## Conclusion

Within the Paraguayan legislation, nine aspects of the 24 sub-recommendations of the WHO framework address the adolescent population. Strategies for education and information provision for adolescents are strongly integrated into legislation, and the government guarantees funding for all types of contraceptives. However, some human rights principles that need to be strengthened in the normative guidance include ensuring non-discrimination, accessibility of quality contraceptive information and services, and community participation. We also note that while more of the recommendations are addressed in the context of all populations, fewer are addressed specifically in the context of adolescents. This could lead to the latter being neglected in actual implementation.

For future modifications or enhancements of the legislation, policy makers may consider taking into account the recommendations of the WHO framework not currently addressed in Paraguay.

## Abbreviations

CSE: Comprehensive Sexuality Education
ECLAC: Economic Commission for Latin America and the Caribbean
FP: Family Planning
GBV: Gender-Based Violence
HIV: Human Immunodeficiency Virus
ICPD: International Conference on Population and Development
LARC: Long-acting Reversible Contraceptives
LGBTI: Lesbian, Gay, Transgender/Transsexual, Intersex
M&E: Monitoring and Evaluation
MSPBS: Ministerio de Salud Publica y Bienestar Social (Ministry of Health and Social Welfare)
SRH: Sexual and Reproductive Health
STI: Sexually Transmitted Infections
UNFPA: United Nations Population Fund
WHO: World Health Organization

## Footnotes

1 “Ensuring human rights within contraceptive service delivery: implementation guide” is available at: http://apps.who.int/iris/bitstream/10665/102539/1/9789241506748_eng.pdf.
